# Targeted Treatment Options in Mastocytosis

**DOI:** 10.3389/fmed.2017.00110

**Published:** 2017-07-20

**Authors:** Mélanie Vaes, Fleur Samantha Benghiat, Olivier Hermine

**Affiliations:** ^1^Department of Hematology, Université Libre de Bruxelles, Hopital Erasme, Brussels, Belgium; ^2^Department of Hematology, Université Libre de Bruxelles, CHU Tivoli, La Louvière, Belgium; ^3^French Reference Center for Mastocytosis (CEREMAST), Department of Hematology, Necker Children’s Hospital, APHP, Paris, France; ^4^Imagine Institute for Genetic Diseases (INSERM U1163 CNRS ERL 8654), Paris Descartes University, Sorbonne Paris Cité, Paris, France

**Keywords:** systemic mastocytosis, mast cell, KIT, targeted treatment, tyrosine kinase inhibitor

## Abstract

Mastocytosis refers to a heterogeneous group of disorders resulting from the clonal proliferation of abnormal mast cells and their accumulation in the skin (cutaneous mastocytosis when only in the skin, CM) or in various organs (systemic mastocytosis, SM). This leads to a wide variety of clinical manifestations resulting from excessive mediator release in CM and benign forms of SM (indolent SM, ISM) and from tissue mast cell infiltration causing multiorgan dysfunction and failure in more aggressive subtypes (aggressive SM, ASM, or mast cell leukemia). In addition, SM may be associated with hematological neoplasms (AHN). While treatment of ISM primarily aims at symptom management with anti-mediator therapies, cytoreductive and targeted therapies are needed to control the expansion of neoplastic mast cells in advanced forms of SM, in order to improve overall survival. Mast cell accumulation results from a gain-of-function mutation (mostly the D816V mutation) within the KIT tyrosine kinase domain expressed by mast cells and additional genetic and epigenetic mutations may further determine the features of the disease (ASM and AHN). Consequently, tyrosine kinase inhibitors and targeted therapies directed against the oncogenic signaling machinery downstream of KIT are attractive therapeutic approaches. A better understanding of the relative contribution of these genetic and epigenetic events to the molecular pathogenesis of mastocytosis is of particular interest for the development of targeted therapies and therefore to better choose patient subgroups that would best benefit from a given therapeutic strategy.

## Introduction

Mastocytosis refers to a heterogeneous group of disorders characterized by the pathologic accumulation of mast cells in different tissues or organs, predominantly skin, bone marrow, and visceral organs (1).

This rare disorder with its high variety of subtle and non-specific clinical manifestations is a real diagnostic challenge. Its exact incidence and prevalence is unknown, but a recent European retrospective population-based study gives an estimate of 1 case per 10,000 persons (2).

In the 2016 WHO classification, mastocytosis is no longer considered a subcategory of myeloproliferative neoplasms (MPN), but a separate entity in myeloid neoplasms with its distinctive clinical and pathologic features (3). Two major forms of mastocytosis are described: cutaneous mastocytosis (CM) and systemic mastocytosis (SM). CM is the most frequent presentation in children, and in most cases, regresses spontaneously at the puberty onset (4). In contrast, SM more often develops in adults and may persist throughout life (5). SM implies an extracutaneous site involvement, most commonly the bone marrow and the gastrointestinal tract, but lymph nodes, spleen, and liver can also be affected (1). Skin involvement is frequent in the benign form of SM, namely, indolent SM (ISM), whereas rarely present in the life-threatening SM subtypes, aggressive SM (ASM), and mast cell leukemia (MCL) (6). SM can also be associated with a non-mast cell clonal hematological neoplasm (SM-AHN), more often myeloproliferative disorders or myelodysplastic syndromes (7).

## Pathogenesis

Recent advances have been made in the understanding of mastocytosis pathogenesis, paving the way for the development of different targeted treatments.

Mast cells derive from hematopoietic progenitors and express on their surface high levels of tyrosine kinase receptor KIT (CD117) that binds the stem cell factor (SCF), a growth factor essential for their survival, maturation, proliferation, migration, and activation (8). KIT is expressed widely on hematopoietic stem cells and on multipotent progenitor cells, but is then downregulated in all mature lineages, except in the mast cell one. Acquired activating KIT mutations lead to SCF-independent receptor activation and signaling, survival, clonal expansion, and uncontrolled activation in mast cells.

In adults, the most common mutation occurs in the codon 816 and consists of a valine-to-aspartate substitution (9). This D816V mutation is located in the phosphotransferase domain of the receptor and causes conformational change in its juxtamembrane region leading to its dimerization and consequently its constitutive activation.

KIT D816V is detected in >80% of all SM cases (9). More than 20 other KIT mutations have been identified such as V560G, D815K, D816Y, VI816_816, D816F, D816H, and D820G (10–16). Disease phenotype and prognosis is apparently not dependent on the type of mutation encountered (12, 17, 18) but is rather correlated to the KIT D816V allele burden. Indeed, a strong correlation exists between the allele burden of KIT mutant determined by highly sensitive techniques such as allele specific quantitative PCR and neoplastic mast cell load, survival, and prognosis (19, 20).

The effect of KIT mutation on mastocytosis phenotype may also be influenced by the development stage of the mutated cell. Indeed, KIT mutations present in multiple lineages (mast cells, myeloid, and lymphoid lineages) have been associated with more aggressive forms of SM (21). In contrast, mutations affecting committed mast cell progenitors or mature mast cells result in milder forms of the disease (22).

In advanced systemic mastocytosis (ASM, MCL, SM-AHN), epigenetic alterations are believed to play a role in the molecular pathogenesis of SM and are of particular interest as potential therapeutic targets. The next-generation sequencing of 70 patients revealed that the most frequently affected genes were TET2 (47%), SRSF2 (43%), ASXL1 (29%), RUNX1 (23%), JAK2 (16%), N/KRAS (14%), CBL (13%), and EZH2 (10%) (23). These mutations are not specific of SM, as they were also identified in other myeloid neoplasms including MPN/myelodysplasic syndrome (MDS) or MPN. These mutations seem to develop before KIT mutations, in almost all patients (24). Such additional lesions may be co-expressed with KIT D816V in the same cells or subclones but may also be detectable in other myeloid lineages, especially in SM-AHN. The prognostic impact of these mutations has been recently studied. Overall survival was adversely affected by mutation in SRSF2, ASXL1, and RUNX1, and the clinical course was also worsening with the number of mutations in the SRSF2/ASXL1/RUNX1 panel (23). SRSF2 has the worse prognosis, and remains, with ASXL1, an independent poor prognostic factor in multivariate analysis (23). Mutations in the tumor suppressor gene TET2 act in synergy with KIT D816V mutation, enhance its oncogenic potency, and induce aggressiveness of the mastocytosis (25), but in contrast to other mutations have not been associated with decreased overall survival (26–28).

Downstream of the phosphotransferase domain mutated KIT, the Jak/Stat5 pathway, and to a less extent the PI3K–AKT signaling cascade are essential for neoplastic mast cells development and proliferation (29) and offers a panel of targeted treatment possibilities, as will be discussed further below.

In childhood, CM is also associated with germline or acquired activating KIT mutations, signing a clonal disease. Available data suggest that approximately 40 percent of children with CM have exon 17 mutation, with another 40 percent carrying KIT mutations outside of exon 17 (17, 30). Some familial mutations of KIT also have been identified, in rare cases of familial mastocytosis (31–33). In contrast to somatic KIT mutations in mastocytosis that were mainly found in exon 17, germline KIT mutations are located in exons 8, 9, 10, 13, and 17 (34). *In vitro*, all these mutations are oncogenic (in contrast of those of the exon 17) and are inducing mainly the PI3-AKT and MAP kinase pathways.

## Clinical Manifestations, Diagnosis, and Classification

Clinical manifestations depend on the subtype of mastocytosis and can be divided in three non-exclusive categories.

### Cutaneous Lesions due to Skin Involvement

Three major variants of CM have been defined by the 2016 WHO classification (see Table 1), the most frequent one being maculopapular mastocytosis (also named urticaria pigmentosa, UP) (35, 36). UP consists in reddish-brown macules or slightly raised papules, classically affecting the upper and lower extremities, sometimes the thorax and the abdomen, but rarely the face or other sun exposed areas. The pathognomonic Darier’s sign refers to swelling, itchiness, and redness appearing after scratching an UP lesion and is due to localized release of mast cell mediators (35). Pruritus and flushing can also be triggered by temperature changes, hot showers, emotional stress, spicy food, fever, exercise, friction, and certain drugs (5).

**Table 1:** WHO 2016 mastocytosis classification.

Cutaneous mastocytosis Urticaria pigmentosa or maculopapular cutaneous mastocytosis Diffuse cutaneous mastocytosis Solitary mastocytoma of skin Systemic mastocytosis Indolent systemic mastocytosis Smoldering systemic mastocytosis Systemic mastocytosis with an associated hematological neoplasm Aggressive systemic mastocytosis Mast cell leukemia Mast cell sarcoma

### Symptoms Associated With Mast Cell Mediator Release

Mediator-related symptoms are a constellation of non-specific signs making the clinical diagnosis of mastocytosis very challenging. These include fatigue, nausea, vomiting, abdominal pain, diarrhea, anaphylaxis, hypotension, diffuse musculoskeletal pain, osteopenia, and osteoporosis (37, 38). Abnormal mast cell degranulation may also occur in the central nervous system leading to psychiatric symptoms such as depression, anxiety, and cognitive impairment (39). More specifically, patients with mastocytosis are more prone to anaphylaxis during allergic reactions, particularly in response to hymenoptera stings (40).

Importantly, in these indolent forms, osteopenia and osteoporosis may occur leading to bone fractures (41, 42). All these symptoms are prominent in smoldering SM (SSM) and ISM while seldom present in ASM.

### Symptoms Related to Organ Infiltration (Only Present in SM)

In advanced SM (ASM, SM associated with another hematological neoplasm and mast cell leukemia), organ damage or dysfunction due to organ infiltration with neoplastic mast cells can include cytopenias, hepatosplenomegaly, portal hypertension, lymphadenopathy, impairment of liver function, ascites, hypersplenism, malabsorption, weight loss, and pathological lytic bone fractures (which must be differentiated from those associated with osteoporosis) (1, 43). The classification of these symptoms in B findings (for “Borderline Benign,” that reflects the disease burden), or C findings (for “require Cytoreductive therapy”), reflecting organ dysfunction and disease aggressiveness, helps to define the subcategory and severity of SM (44) (see Table 2).

**Table 2:** B and C findings.

“B” findings	“C” findings
BM biopsy showing >30% infiltration by MC (focal, dense aggregates) and/or serum total tryptase level >200 ng/mL Signs of dysplasia or myeloproliferation, in non-MC lineage(s), but insufficient criteria for definitive diagnosis of a hematopoietic neoplasm (AHN), with normal or slightly abnormal blood counts. Hepatomegaly without impairment of liver function, and/or palpable splenomegaly without hypersplenism, and/or lymphadenopathy on palpation or imaging	Bone marrow dysfunction manifested by one or more cytopenia(s) (ANC < 1.0 × 10^9^/L, Hb < 10 g/dL, or platelets < 100 × 10^9^/L), but no obvious non-mast cell hematopoietic malignancy. Palpable hepatomegaly with impairment of liver function, ascites, and/or portal hypertension. Skeletal involvement with large osteolytic lesions and/or pathological fractures. Palpable splenomegaly with hypersplenism. Malabsorption with weight loss due to gastrointestinal mast cell infiltrates.

*BM, bone marrow; MC, mast cell; ANC, absolute neutrophil count; Hb, hemoglobin*.

Systemic mastocytosis diagnosis, according to the WHO classification, requires the presence of both the major criterion and one minor, or at least three minor criteria (1). The major criterion is defined as the presence of multifocal, dense infiltrates of mast cells (>15 mast cells in aggregates) detected in bone marrow and/or other extracutaneous organs. Minor criteria are the following: (1) >25% of mast cells in infiltrates with atypical morphology; (2) detection of an activating point mutation at codon 816 of KIT in bone marrow, blood, or an extracutaneous organ; (3) mast cells abnormal expression of CD2 and/or CD25; and (4) serum total tryptase level >20 ng/ml.

The 2016 WHO classification recognizes further five subtypes of SM in order to stratify mast cell disorders according to their aggressiveness (3):

– ISM, displaying no evidence of extracutaneous organ dysfunction;
– SSM, defined by the presence of two or more B findings (see Table 2);
– Aggressive systemic mastocytosis (ASM), defined by the presence of one or more C findings (see Table 2);
– SM associated with another hematological neoplasm (SM-AHN);
– Mast cell leukemia (MCL), defined by >20% mast cells on bone marrow smear or >10% in peripheral blood.

Systemic mastocytosis prognosis differs according to the disease subtype and will subsequently guide treatment strategy. A study of 342 patients with SM of the Mayo clinic showed that ISM survival is comparable to age-and sex-matched control population, whereas advanced SM patients clearly have a significantly inferior survival with a median of 41 months for ASM, 24 months for SM-AHN, and 2 months for MCL (45). Recently, a form of chronic MCL with a low index of proliferation (Ki67) has been described with a better prognosis (46).

## Treatment of Advanced SM

**Figure 1:**
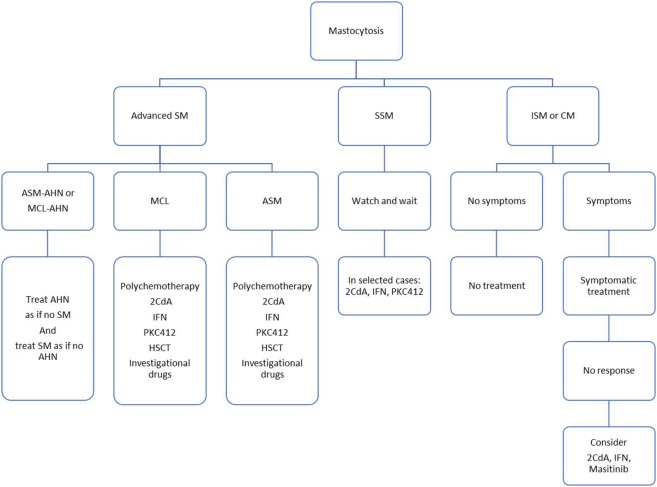
Proposed treatment algorithm.

A proposed treatment algorithm is presented in Figure 1. Various cytoreductive treatments have been used for advanced SM, including 2-Chlorodeoxyadenosine (2-CDA), interferon-alpha (IFN-α), classical chemotherapy agents (such as cytarabine or fludarabine), but all with modest and disappointing response rates, highlighting the need for innovative therapies (47, 48).

### Cytoreductive Therapies

For many years, IFN-α has been considered as the first line treatment for patients with advanced mastocytosis, but the efficacy was variable, and the exact posology and treatment duration remained unknown. In the Mayo Clinic study published in 2009, the overall response rate (ORR) in 40 IFN-α-treated patients was 53% with only 1 complete response (CR), 6 major responses (MR), and 14 partial responses (PR), and a median duration of response of 12 months (range 1–67 months) (49). Major toxicities were observed, including fatigue, depression, and thrombocytopenia. Interestingly, IFN-α has also a role in treating skeletal symptoms because of its ability to increase bone density. The median weekly dose was 15 million units/week, ranging from 0.5 to 10 MU three times a week (49).

Cladribine (or 2-CdA) has shown therapeutic activity in all SM subtypes, including MCL. In the Mayo Clinic study, the ORR in 22 cladribine-treated patients was 55% (CR 5%, MR 32%, and PR 18%), with a mean duration of response of 11 months (range 3–74). Major toxicities included myelosuppression and infections (49). Improved response rates were observed in a recent French study on 68 patients (36 with ISM and 32 with advanced SM) treated with 2-CdA with 72% ORR, 92% in ISM (by reducing symptoms and skin involvement), and 50% in advanced SM and a median duration of response of 3.7 and 2.47 years for ISM and advanced SM, respectively. The administered dose was 0.14 mg/kg intravenous or subcutaneously for 5 days, repeated at 4–12 weeks, with a median number of cycles of 3.7 (range 1–9) (50). As expected, major toxicities were leukopenia and opportunistic infections.

### Tyrosine Kinase Inhibitors

Tyrosine kinase inhibitors (TKIs) are an attractive therapeutic approach, given the pathogenesis of SM and the involvement of KITD816V mutation in more than 80% of patients, and other KIT mutations that map to the TK juxtamembrane domain or transmembrane domain in sporadic cases of SM (48).

#### Imatinib

Imatinib is an efficient inhibitor of wild-type KIT, PDGFR, and BCR-ABL, but has no activity against the KITD816V mutation (51). Indeed, this mutation induces structural alterations at the KIT binding site, resulting in a decreased affinity for type I TKIs, such as imatinib, that recognize the active conformation of the kinase (52). Consequently, imatinib failed to demonstrate any response in KIT D816V mutated SM (53). However, imatinib may be an appropriate candidate in the rare SM cases that display an imatinib-sensitive KIT mutation (F522C, K5091, V560G, V559G, and del419) (54, 55), or those without the KITD816V mutation (56, 57). For this reason, the US Food and Drug Administration approved imatinib only for ASM patients not harboring the KITD816V mutation or ASM patients with an unknown mutational status.

The difficult distinction between certain forms of hypereosinophilic syndrome (HES) and SM has contributed to the recommendation of imatinib as therapy for mast cell disease with hypereosinophilia in earlier reports. Indeed, mast cells and eosinophils may be found in both disorders; however, when present, genetic mutations (KIT mutations and FIP1L1-PDGFRa rearrangement) are the diseases distinctive signature (58). Only a few patients carry both KITD816V and FIP1L1-PDGFRa rearrangement (59). The delineation between FIP1L1-PDGFRa HES and KIT D816V advanced SM with eosinophilia has important clinical implications, as those with FIP1L1-PDGFRa rearrangement respond to imatinib and not the others (60).

#### Dasatinib

Dasatinib is a multikinase inhibitor active against BCR-ABL1, KIT, and PDGFRa and has shown promising *in vitro* activity against various KIT mutants, including D816V (61, 62), but its very short half-life *in vivo* may be responsible for the disappointing clinical response. In the largest phase 2 study of dasatinib in SM (33 patients, 15 with advanced SM), 2 (6%) of the 33 patients achieved CR and 9 (27%) achieved a symptomatic improvement. ORR was 33% but 58% experienced grade 3 toxicities, mainly pleural effusions and thrombocytopenia (63). In view of these elements, dasatinib is nowadays not recommended in the treatment of advanced SM patients.

#### Nilotinib

Nilotinib has been investigated in a phase 2 trial with 61 patients (including 37 with advanced SM), at the dose of 400 mg twice daily. The ORR was 21.6% overall and 21% in advanced SM (64). Regarding to its modest activity, nilotinib has currently no place in the treatment of SM.

#### Bosutinib

Bosutinib is a dual SRC/ABL kinase inhibitor, with minimal anti-KIT activity. *In vitro*, bosutinib is able to decrease neoplastic mast cell growth by inhibiting LYN and BTK activity (65). However, no clinical response has been shown in a patient with ASM treated with bosutinib (66).

#### Ponatinib

Ponatinib, another multikinase inhibitor, has shown activity on KITV560G and, less effectively, on KITD816V in the human mast cell leukemia cell line human mast cell line-1 (HMC-1) (67, 68). Ponatinib also synergizes with midostaurin to obtain growth inhibition against neoplastic mast cells harboring the KITD816V mutant (67). However, clinical trials are needed to assess the *in vivo* efficacy of ponatinib, alone or in combination.

#### Masitinib (AB1010)

Masitinib (AB1010) is a KIT inhibitor with activity against KIT and LYN kinases, but with no activity on KITD816V mutants (69). Few anecdotal cases with aggressive forms bearing KIT mutations outside exon 17 or no KIT mutation have responded durably (70). Its emerging role in the treatment of indolent mastocytosis will be discussed further below.

#### Midostaurin (PKC412)

Midostaurin (PKC412) is an oral potent multikinase inhibitor with activity against protein kinase C (PKC), FMS-related tyrosine kinase 3 (FLT3), PDGFRA/B, vascular endothelial growth factor receptor 2, and KIT (71). Interestingly, midostaurin shows clinical activity and efficacy regardless of the KIT mutation status. In the recently published phase 2 multicenter international study, 116 patients with advanced SM received 100 mg of midostaurin twice daily until progression or unacceptable toxicity (72). Eighty-nine patients were evaluable for efficacy, including 16 with ASM, 57 with SM-AHN, and 16 with MCL. After a median follow-up of 26 months (range 12–54 months), the ORR was 60% with 45% of MR and 15% of PR and the median OS was 28.7 months (72). Responses occurred in multiple organ systems, including resolution of pleural effusions, hypoalbuminemia, reversion of weight loss, improvement in liver function, and increase in hemoglobin and platelet counts. In responding patients, durable responses were observed, with a median duration of response of 24.1 months and a median OS of 44.4 months. Results were similar no matter the KIT mutational status and were similar in different subtypes of advanced SM. In the 16 patients with the highly aggressive MCL subtype, the ORR was 50%, 7 patients experienced MR (44%). Their median OS was 9.4 months overall, but median OS in responders has not been reached. A significant (>50%) decrease in bone marrow MC burden and tryptase levels has also been observed. Toxicities included mainly grade 1–2 gastrointestinal adverse events (AEs), and grade 3–4 anemia, neutropenia and thrombocytopenia was observed in 41, 24, and 29%. respectively, mainly in patients with preexisting cytopenias. Sixty-five patients (56%) needed dose reduction, mainly because of AEs, with possible reescalation to the initial dose in 21 of the 65 patients (32%). Midostaurin has therefore a favorable efficacy and safety profile. It can induce durable responses in patients with advanced SM, even in MCL patients, and should be considered as a part of the first-line treatment in advanced SM.

#### BLU-285

BLU-285, a potent and selective KITD816V inhibitor, has shown encouraging results in preclinical studies (73) but also in an ongoing phase I trial. So far, 12 patients with advanced SM have been treated with BLU-285 at three dose levels (30, 60, or 100 mg once daily) (74). Eleven of the 12 patients harbored the KIT D816V mutation. BLU-285 appeared to be well tolerated at all doses since no patients discontinued treatment due to AEs, and no grade ≥ 4 AEs were reported. The majority of the AEs were grade 1 or 2 and included fatigue, dizziness, headache, rash, shingles, anemia, and thrombocytopenia (*n* = 1 for each). Objective decreases in mast cell burden were observed in six out of eight evaluable patients, including decline in peripheral blood and BM KIT D816V DNA levels. Serum tryptase levels declined in 10 out of 12 patients (83%), and half of the patients experienced a decrease in BM infiltrate. Symptomatic improvement was also reported with less allergy symptoms, improved UP and increased albumin, and weight gain (74).

### Antibody-Mediated Targeted Therapy

Normal and neoplastic mast cells express on their surface a number of cell surface antigens that might be considered as potential targeted therapies in advanced SM, some of them being already available and used in other hematological diseases. These antigens include CD13, CD25, CD30, CD33, CD44, CD52, CD87, CD117, and CD123 (75).

In contrast to normal mast cells, neoplastic mast cells abundantly express the cell-membrane protein CD30 on their surface but also in their cytoplasm (76, 77). As assessed by flow cytometry, CD30 was found on neoplastic mast cells in 12% of patients with ISM and 57% of patients with ASM or MCL, making it an attractive target for advanced SM (78). Brentuximab vedotin (a CD30-targeted antibody conjugated with the antimitotic agent auristatin E) is already an established treatment for Hodgkin lymphoma and anaplastic large cell lymphoma. In patients with CD30(+) SM, brentuximab vedotin induces apoptosis of neoplastic MCs, downregulates IgE-mediated histamine release in CD30(+) MCs, and synergizes with midostaurin to inhibit neoplastic MC growth (78). In a small case series of four patients with ASM or ISM, brentuximab vedotin led to a reduction in the disease burden in half of them, of which one experienced a durable response for more than 3 years (79). Besides, treatment with brentuximab vedotin was well tolerated with toxicities manageable by dose reduction only. Together, CD30 appears to be a promising new drug target for patients with CD30(+) advanced SM, with a favorable toxicity profile. Further studies are needed to determine its efficacy and potential combination with midostaurin.

CD52 is another potential target widely expressed on the surface of neoplastic MCs mainly in advanced SM (80). The CD52-targeted antibody alemtuzumab has been shown to induce neoplastic mast cell death *in vitro* but also *in vivo*, in xenotransplanted mice with HMC-1. So far, no clinical studies have been performed.

In a similar way, gemtuzumab ozogamicin (Mylotarg^®^), an anti-CD33 monoclonal antibody conjugated with a cytostatic agent, can also induce cell death in neoplastic MCs and their progenitors *in vitro* (81). We have treated a patient with MCL, who was refractory to all previous treatment (cladribine, midostaurin, chemotherapy), with a MR allowing bone marrow transplantation (O. Hermine, personal observation).

CD123, the α-subunit of the interleukin-3 receptor, represents also a potential therapeutic target as it is aberrantly expressed on neoplastic MCs and absent on normal MCs (82, 83). Clinical trials are ongoing to evaluate its efficacy in patients with SM.

More recently, it has been shown that mast cells in mastocytosis may express PD-L1, suggesting that effects of checkpoint inhibitor antibodies should be tested in clinical trials in this disease (84).

### Targets Related to Signaling or Apoptosis

Several studies have reported quantitative and qualitative defects of signal transduction in SM. These altered pathways play a role in the pathogenesis of SM and targeted drugs may provide therapeutic options by selective inhibition of some of these critical pathways.

Neoplastic MCs development seems to be essentially governed by the STAT5–PI3K–AKT–mTOR signaling cascade downstream of the mutated KIT (85, 86). PI3K (phosphoinositide 3-kinase), a lipid kinase, is important for the function of intracellular signaling molecules, like BTK, AKT and PDK1. Mutated KIT constitutively activates PI3K, which in turn phosphorylates AKT and subsequently mTOR, promoting abnormal mast cell development *in vivo* and *in vitro* (86). Activated KIT also recruits the JAK/STAT signaling pathway and STAT5 especially (87). Small inhibitor molecules targeting STAT5 or AKT might therefore be of particular interest in treating patients with SM. Unfortunately to date, AKT inhibitors have shown efficacy only *in vitro*, and STAT5 targeting drugs can be effective only at high concentrations *in vivo* (48).

mTOR, a conserved Serine/Threonine kinase, exists in two distinct multimolecular complexes: mTOR complex1 and mTOR complex2. Expression and activation of mTORC1 and mTORC2 is increased in neoplastic human MC lines and in immature normal MCs, compared with mature normal MCs (88). Rapamycin has shown to specifically block mTORC1 in normal MCs and to inhibit cell survival of tumor mast cells bearing the C-KIT D816V mutation (89). In contrast, everolimus, another mTOR inhibitor, was found ineffective in patients with SM (90). We have treated a patient with an ASM refractory to cladribine and midostaurin, who responded to the combination of temsirolimus and high-dose aracytine, with a MR and who is now cured 2 years after an allogeneous stem cell transplantation. BEZ235, a dual PI3K/mTOR blocker, produces growth-inhibitory effects in immature neoplastic MC and inhibits IgE-dependent activation of mature basophils and MCs (91). Whether these potentially beneficial drug effects have clinical implications is currently under investigation.

Bim, a proapoptotic Bcl-2 family member, is downregulated by KITD816V and has been identified as a tumor suppressor in neoplastic mast cells (92). Midostaurin and the proteasome-inhibitor Bortezomib enhance the expression of Bim in MC leukemia cell lines HMC-1.1 (D816V negative) and HMC-1.2 (D816V positive), decreasing their survival. Furthermore, midostaurin was found to cooperate with bortezomib and with the pan-Bcl2 family inhibitor obatoclax in reducing proliferation and survival in both HMC-1 subclones (92). Targeting Bcl-2 family members by drugs promoting Bim (re)-expression, or by BH3-mimetics such as obatoclax, may be an attractive therapy concept in SM.

MCL-1, a BCL-2 family member with antiapoptotic properties, is expressed in neoplastic MCs (93). MCL-1 inhibition with antisense oligonucleotides increased apoptosis in these cell lines, and increased responsiveness to TKI such as midostaurin, suggesting a novel interesting target that could help overcome resistance to TKI.

NF-kB and NFAT (nuclear factor of activated T cells), two transcription factors of the REL family, have been found to be constitutively activated in KIT mutated cells and could also represent interesting targets (94, 95). Indeed, by inhibiting NF-kB activity with IMD-0354, HMC-1 cells spontaneous proliferation was completely repressed (94). Similarly, in *in vitro* assays on KIT mutated mast cell lines, combining a KIT inhibitor with a NFAT-regulator such as a calcineurin phosphatase inhibitor, leads to synergistic increase in cell apoptosis (95).

More recently, the epigenetic reader bromodomain-containing protein-4 (BRD4) has been identified as a novel potential target, as neoplastic MCs express substantial amounts of BRD4 in ASM and MCL (96). The BRD4-targeting drug JQ1 (a drug blocking the specific interactions between BRD4 and acetylated histones) induces dose-dependent growth inhibition and apoptosis in primary neoplastic cells obtained from patients with advanced SM as well as in HMC-1 and ROSA cells (96). Interestingly, drug effects could be potentiated by addition of PKC412 or ATRA (all trans retinoic acids). Whether these targeted drugs are effective *in vivo* has yet to be determined.

Histone deacetylase inhibitors (HDACi) may also be of clinical interest for treatment of SM. In particular, suberoyl anilide hydroxamic acid (SAHA), also known as vorinostat, have been shown to induce apoptotic cell death in mast cell lines as well as in MCs from patients with SM, through a specific epigenetic downregulation of KIT, whereas healthy bone marrow MCs are less sensitive (97). The HDACi AR-42 has also been described to downregulate constitutively active KIT in malignant murine and canine MCs (98).

### Allogeneic Hematopoietic Stem Cell Transplantation (AlloHSCT)

As currently available treatment options fail to achieve durable remissions, alloHSCT remains the only potentially curative treatment for patients with advanced SM and has to be considered in those patients. In the largest published case series, 57 patients received stem cell transplant, mostly from HLA-identical (*n* = 34) or unrelated donors (*n* = 17), and with myeloablative conditioning (*n* = 36) or reduced-intensity conditioning (*n* = 21) (99). Overall survival was 57% at 3 years for all patients, 74% for patients with SM-AHNMD, 43 and 17% for those with ASM and MCL, respectively. The strongest risk factor for poor OS was a diagnosis of MCL (99). Consensus opinion on HSCT in advanced SM and consensus criteria of treatment response were recently published in order to help standardize assessment of treatment response and optimal management in this rare, heterogenous, and severe disease (100, 101).

### Treatment for SM Associated With Another Hematological Neoplasm (SM-AHN)

In any variant of SM, another associated hematological neoplasm may be diagnosed as a concomitant disease. As for SM, the AHN component has to be determined by WHO criteria. In most of the patients with AHN, a myeloid neoplasm is diagnosed: chronic myelomonocytic leukemia is commonly detected, but also acute myeloid leukemia (AML), JAK2-mutated myeloproliferative neoplasms (MPN), or MDS overlap disorders. Lymphoproliferative disorders (myelomas and lymphomas) have also been reported, however, more rarely. Coexistence of SM with Philadelphia positive chronic myeloid leukemia is an extremely rare condition. The SM component in SM-AHNMD often presents as ASM and less frequently as MCL. The standard recommendation is to treat the SM component of the disease as if no AHN was diagnosed and to treat the AHN component as if no SM was found, with special attention to potential drug interactions and side effects (44). Patients with ASM-MDS for example could be successfully treated with hypomethylated agents like azacitidine in combination with midostaurin (102). In a similar way, ASM or MCL with associated AML should be treated with midostaurin combined to high-dose chemotherapy, with consideration for allogeneic stem cell transplantation if a certain degree of response is obtained. Moreover, recent data showed that high-risk hematologic neoplasms such as FLT3-positive AML can be managed effectively with midostaurin in combination with chemotherapy (103). Interestingly, in AML associated with ASM, even patients not in CR could be transplanted with a favorable outcome. Finally, patients with an associated myeloproliferative neoplasm exhibiting JAK2 mutations or a JAK2 fusion gene product can be responsive to JAK2-targeting drugs, such as ruxolitinib.

## Treatment for ISM and SSM

Treatment for ISM is based on mediator-related symptom management, as they are likely to have a normal life expectancy. The keystone of the treatment is to recognize and avoid triggers of MC degranulation. These often are food, stress, excessive heat or cold, hymenoptera stings, alcohol (red wine), and medications as non-steroidal anti-inflammatory drugs, aspirin, or opioids (104).

Most patients with ISM respond to a combination of H1- and H2-histamine receptor antagonists, the standard therapy for pruritus and flushing, and abdominal pain, cramping, and diarrhea, respectively. In patients with persistent gastrointestinal symptoms, adding a proton pump inhibitor may be beneficial in combination with anti-H2 drugs. Cromolyn sodium, a MC stabilizer, can also be useful if gastrointestinal symptoms control is insufficient (105). Adding leukotriene antagonists may be useful, particularly in recalcitrant skin symptoms (106). When conservative measures are unsatisfactory, short courses of corticosteroids may be required to curb refractory symptoms (48, 104). Finally, some patients refractory to optimal conventional therapy will require mast cell cytoreductive treatments, mainly 2-CdA or IFNα (104). In those cases, a careful evaluation of the handicap linked to the symptoms is critical to weigh the beneficial/risk ratio of cytoreductive treatment in ISM.

In patients with SM, special attention has to been made for osteoporosis. Indeed, a cohort study of 75 patients with SM revealed that osteoporosis was present in 31% of patients (42). Osteoporosis should be screened actively and treated with bisphosphonates if indicated. In case of resistant osteoporosis or intolerance to bisphosphonate, alternative drugs may be considered, including low-dose IFN-α or RANKL inhibitors such as denosumab.

Prognosis and natural clinical course of patients with SSM has not been clearly defined, but risk of disease progression and leukemic transformation may be higher and survival shorter than in ISM (107). However, according to general recommendations, patients with SSM who have no symptoms or signs of progression do not require any specific therapy (47, 104). In case of mediator-related symptoms, treatment is identical to that of ISM. In SSM patients with severe anaphylaxis or signs of progression, 2-CdA or IFN is often recommended and is usually effective in reducing the MC burden.

*Masitinib (AB1010)* is a KIT inhibitor with activity against wild-type KIT, PDGFR, and Lyn, but with no activity on KITD816V mutants (69). Nevertheless, in a phase 2 study in 25 patients with CM or ISM harboring symptoms refractory to conventional therapy, masitinib showed a significant improvement in the frequency of flushing (62%), pruritus score (36%), and Hamilton rating for depression (43%) (108). The overall clinical response, defined as >50% improvement in baseline symptom, was 56% and maintained at 60 weeks. Toxicity profile was acceptable, with mostly nausea and vomiting (52%), edema (44%), muscle spasms (28%), and rash (28%); however, one patient experienced reversible agranulocytosis (108). In a recently published phase 3 randomized trial, 135 patients were randomized to receive either masitinib (6 mg/kg/day over 24 weeks with possible extension) or placebo (109). By 24 weeks, masitinib was associated with a cumulative response (i.e., >75% improvement from baseline within weeks 8–24) of 18.7% compared with 7.4% for placebo for the following symptoms: pruritus, flushes, depression according to Hamilton scale, and severe fatigue (109). The most frequent severe AEs included diarrhea (11%), rash (6%), and asthenia (6%). Surprisingly, with time, mast cell burden decreases with tryptase level reduction and improvement of skin lesions. These studies indicate that masitinib provides symptomatic improvement in ISM or SSM with severe symptoms refractory to conventional treatment, with an acceptable toxicity profile. Its effects might be due to the inhibition of WT C-KIT, FYN, and LYN, which participates to mast cell activation.

### Management of Allergy in Mastocytosis

Prevalence of allergy and atopic disorders in patients with mastocytosis is identical to that of the general population (110–112), but incidence of anaphylaxis is significantly higher, and ranges from 20 to 49% (112–114). Anaphylaxis in mastocytosis may be IgE-mediated or IgE-independent without any identified triggers and is more likely to manifest itself with hypotension as well as life-threatening circulatory collapse. Therefore, some authors recommend the prescription of an epinephrine pen for patients with coexisting allergies, in case of the acute onset of severe symptoms of anaphylaxis (47, 104). Major triggers are Hymenoptera stings, foods, and medications, although in approximately 40%, the elicitor is not known (115). Besides, patients with detectable IgE against bee or wasp venom should undergo life-long hymenoptera venom immunotherapy (116).

In some patients with refractory symptoms of allergy, with high risk of life-threatening anaphylaxis, antibody-mediated depletion of IgE with omalizumab may be useful (117). This humanized IgG kappa monoclonal antibody against IgE has shown a reduction in the frequency of anaphylaxis in limited case studies (117–121). It can also be used in patients with daily symptoms whose disease has been unresponsive to classical treatment, even if the underlying mechanism is not completely understood. Omalizumab inhibits IgE binding to the surface of mast cells and basophils by forming complexes with free IgE in the serum. This triggers the downregulation of the high-affinity IgE receptor (FcepsilonRI) expression on mast cells and basophils and subsequently the reduction of mast cell activation and reactivity (122) However, omalizumab does not seem to decrease mast cell burden, as reflected by stable serum tryptase levels during the treatment (118).

## Conclusion

During the last two decades, major discoveries have been made for better diagnosis, identification of the clinical and biological abnormalities, and for a better classification of the different forms of mast cell disease, allowing now a better prognosis stratification. In addition, the knowledge of molecular pathways involved in the pathophysiology of mastocytosis has led to the emergence of new symptomatic and cytoreductive drugs that have dramatically improved the quality of life and survival of patients with mastocytosis. However, progress is still needed particularly for controlling psychiatric and neurological symptoms in ISM and to decipher molecular pathways involved in ASM, MCL, and sarcoma in the hope to find new targeted drugs or to use new combination.

## Author Contributions

MV, FB, and OH: design of the review. MV: manuscript writing. FB and OH: critical revision and final approval.

## Conflict of Interest Statement

OH: AB Science Cofounder, Stockholder, Research grants Novartis Research grants. All other authors have no conflicts of interest to declare.
